# The Horace Wells Permanent Memorial

**Published:** 1895-05

**Authors:** J. D. Thomas

**Affiliations:** Chairman of the Central Executive Committee


### The Horace Wells Permanent Memorial,
UNDER THE AUSPICES OF THE AMERICAN DENTAL ASSOCIATION.

*To the Dental Profession of America:*

The Central Executive Committee appointed by the President of the American Dental Association is as follows: Dr. James Truman, Dr. Wilbur F. Litch, Dr. S. H. Guilford, Dr. E. C. Kirk, Dr. J. D. Thomas, Chairman and Treasurer.

This committee has been completed in its organization by including in its membership the presidents of all dental societies throughout the United States.

It is hoped to secure enough money to erect a bronze statue of Horace Wells in the national capital. The details as the style and character of the statue, as well as its definite location, will be decided upon at the next meeting of the American Dental Association, to be held at Asbury Park, N. J.

The committee takes pleasure in calling your attention to this opportunity for doing an act of justice to the memory of a worthy member of our profession, whose discovery has been of such incalculable benefit to humanity, and which has been so great an honor to our profession. You are invited to contribute whatever amount of money you may feel able and willing to donate to the fund, and to use your influence toward bringing our plan to a successful issue in a manner befitting the object.

Contributions may be sent by any member of the profession through the president of his local society, or direct to the treasurer, Dr. J. D. Thomas, 912 Walnut street, Philadelphia. An official receipt will be issued by the treasurer for all contributions. The full list of contributors will be embodied in the pedestal of the memorial.

J. D. THOMAS,
*Chairman of the Central Executive Committee.*

---

The Executive Committee requests that all who desire copies of the souvenir volume of the meeting held at Philadelphia, December 11, 1894, in celebration of the fiftieth anniversary of the discovery of anæsthesia by Horace Wells, will promptly forward their names to the chairman, in order that the number of copies to be printed may be determined upon. The price has been fixed at $1.50 per volume, postage free to all parts of the United States; foreign countries at regular postal rates.

